# Nigella and Milk Thistle Seed Oils: Potential Cytoprotective Effects against 7β-Hydroxycholesterol-Induced Toxicity on SH-SY5Y Cells

**DOI:** 10.3390/biom11060797

**Published:** 2021-05-27

**Authors:** Souha Hammouda, Imen Ghzaiel, Pol Picón-Pagès, Wiem Meddeb, Wided Khamlaoui, Sonia Hammami, Francisco J. Muñoz, Mohamed Hammami, Amira Zarrouk

**Affiliations:** 1 Biochemistry Laboratory, LR12ES05 Nutrition-Functional Foods and Vascular Health, Faculty of Medicine, University of Monastir, Monastir 5019, Tunisia; souhahammouda51@gmail.com (S.H.); imenghzaiel93@gmail.com (I.G.); wided.92.92@gmail.com (W.K.); sonia.hammami@fmm.rnu.tn (S.H.); mohamed.hammami@fmm.rnu.tn (M.H.); 2 Laboratory of Molecular Physiology, Faculty of Health and Life Sciences, Universitat Pompeu Fabra, 08003 Barcelona, Spain; pol.picon@upf.edu (P.P.-P.); paco.munoz@upf.edu (F.J.M.); 3 Faculty of Sciences, University of Carthage, Bizerte 7021, Tunisia; wiem.meddeb@gmail.com; 4 Faculty of Medicine, Sousse, University of Sousse, Sousse 4000, Tunisia

**Keywords:** 7β-hydroxycholesterol, neuroblastoma cells, cellular oxidative stress, neurodegenertion, antioxidants enzymes, antioxidants

## Abstract

Oxysterols are assumed to be the driving force behind numerous neurodegenerative diseases. In this work, we aimed to study the ability of 7β-hydroxycholesterol (7β-OHC) to trigger oxidative stress and cell death in human neuroblastoma cells (SH-SY5Y) then the capacity of Nigella sativa and Milk thistle seed oils (NSO and MTSO, respectively) to oppose 7β-OHC-induced side effects. The impact of 7β-OHC, associated or not with NSO or MTSO, was studied on different criteria: cell viability; redox status, and apoptosis. Oxidative stress was assessed through the intracellular reactive oxygen species (ROS) production, levels of enzymatic and non-enzymatic antioxidants, lipid, and protein oxidation products. Our results indicate that 7β-OHC (40 µg/mL) exhibit pr-oxidative and pro-apoptotic activities shown by a decrease of the antioxidant enzymatic activities and an increase of ROS production, lipid, and protein oxidation end products as well as nitrotyrosine formation and caspase 3 activation. However, under the pre-treatment with NSO, and especially with MTSO (100 µg/mL), a marked attenuation of oxidative damages was observed. Our study suggests harmful effects of 7β-OHC consisting of pro-oxidative, anti-proliferative, and pro-apoptotic activities that may contribute to neurodegeneration. NSO and especially MTSO showed potential cytoprotection against the cytotoxicity of 7β-OHC.

## 1. Introduction

The involvement of lipid metabolism disorders in neurodegenerative diseases is widely reported [1,2,3,4].

Cholesterol oxides, also named oxysterols, are 27-carbon intermediates or end-product of cholesterol metabolism. These molecules can be brought by the diet, formed by auto-oxidation and/or enzymatic processes. Some oxysterols, such as 7-ketocholesterol (7KC) and 7β-hydroxycholesterol (7β-OHC), exhibit cytotoxic and pro-inflammatory activities, which are characteristics of several chronic diseases and age-related diseases [2,5,6]. Unlike cholesterol, oxysterols are also able to cross the blood–brain barrier. Thus, a growing interest in elucidating their implication in the development of neurodegenerative diseases has emerged.

As the brain is rich in cholesterol [7], this makes it vulnerable to reactive oxygen species (ROS) attack with subsequently oxysterols’ formation, such as 7β-OHC. In Parkinson’s disease (PD), an accumulation of 7β-OHC was observed in the visual cortex [8]. Likewise, high levels of 7β-OHC were detected in the putamen in Huntington’s disease (HD) [9]. In Alzheimer’s disease (AD) condition, an association of high levels of 7β-OHC with oxidative stress biomarkers was reported in plasma of AD patients [4]. It is noteworthy that AD is characterized by the accumulation of amyloid-beta (Aβ), produced from the amyloid precursor protein (APP) via a proteolysis reaction. Nelson and Alkon reported that both Aβ and APP contribute to the enhancement of the neurotoxic effects of 7β-OHC [10]. In vitro, 7β-OHC was found to promote oxiapoptophagy in different cell lines, which is a mixed-mode of cell death associated with oxidative stress, apoptosis, and autophagy. Furthermore, this molecule induced modifications of lipid profile as well as mitochondria, lysosome, and peroxisome dysfunctions [11]. 7β-OHC was also associated with Aβ generation and neuron death [10]. In cultured rat hippocampal neuronal cells, the APP induced the oxidation of cholesterol to 7β-OHC [10]. All this evidence supports the contribution of oxysterols, in particular 7β-OHC, in neurodegenerative diseases through oxidative stress. Thus, identifying natural products preventing or attenuating the oxysterol-induced deleterious effects could be of interest.

Antioxidant therapy is believed to be an essential tool in the prevention and treatment of several oxidative stress-related diseases [12,13]. In traditional medicine, vegetable oils constitute an important source of bioactive molecules such as tocopherols and polyphenols, which exhibit potent neuroprotective properties and can cross the blood–brain barrier [14,15,16]. In the present study, our interest has been given to milk thistle (Silybum marianum) and nigella (Nigella sativa), plants known to have antioxidant properties. 

In the literature, several researchers pointed out to Nigella seeds oil (NSO) anti-inflammatory and antioxidant activities [17,18,19]. The biological activity of this oil was associated not only to scavenge free radicals but also to modulate the endogenous antioxidant systems. Among the compounds in NSO, thymoquinone is known as a potent antioxidant [20,21]. Milk thistle seeds oil (MTSO) is also widely known in phytotherapy especially in the treatment of liver diseases. This oil contains several bioactive components including antioxidants, tocopherols, and carotenoids which may explain its therapeutic effect [22]. In vitro, milk thistle was found to promote differentiation and neuronal survival [23]. Besides, it protects rat hippocampus neurons against cell death [23].

In this line and based on the literature, we aimed to study the capacity of 7β-OHC to trigger cellular modifications involved in the neurodegenerative diseases such as oxidative stress and cell death on human neuroblastoma cell line (SH-SY5Y cells) then to evaluate the antioxidant properties of NSO and MTSO and their cytoprotective capacity against 7β-OHC-induced side effects.

Overall, our results indicate that 7β-OHC induce deleterious effects on SH-SY5Y cells consisting of ROS overproduction, lipid and protein oxidation, loss of antioxidant enzyme activities, and induction of apoptosis. NSO and MTSO attenuated 7β-OHC-induced side effects. A more significant effect was observed with MTSO, though. These findings consolidate the harmful effects of 7β-OHC consisting of pro-oxidative and pro-apoptotic activities which may contribute to neurodegeneration. NSO and especially MTSO showed potential cytoprotection against the cytotoxicity of 7β-OHC.

## 2. Materials and Methods

### 2.1. Chemical Contents and Antioxidants Activities of NSO and MTSO

#### 2.1.1. Seed Oils Preparation

The seeds oils were extracted via cold-pressing by Dr. Wiem Meddeb (Higher institute of Biotechnology, Beja, Tunisia) and stored at (−20 °C) for analysis as previously reported [19].

#### 2.1.2. Determination of Lipid Contents

Lipids were extracted from seed oils as described by Moilanen and Nikkari [24]. C19:0 was added as an internal standard. After transmethylation of lipids with boron trifluoride in methanol, methyl esters were extracted with hexane and then analyzed by gas chromatography using a Hewlett Packard Gas Chromatograph (Hewlett Packard 5890, Palo Alto, CA, USA) with HP-Innovax column (30 m × 0.25 mm 0.25 μm film thickness: Hewlett-Packard, Palo Alto, CA, USA). The identification of the fatty acid methyl esters was carried out following a comparison with synthetic standards (Sigma-Aldrich, France). The results were analyzed with the EZ Chrom Elite software (Agilent Technologies, Massy, France) and data were shown as % of total fatty acids.

#### 2.1.3. Determination of Tocopherol and Polyphenol Contents

Tocopherol analysis was performed as previously described [25]. Briefly, oil samples were resuspended in high-performance liquid chromatography (HPLC) mobile phase comprising acetonitrile/methanol, ammonium acetate/water/dichloromethane (50 mM) (700:150:50:100, *v*/*v*/*v*/*v*), then vortexed for 30 s. For the analysis, 80 μL of each sample were injected into a reverse phase HPLC system.

Polyphenol analysis was performed as described previously [25]. Oil samples were dissolved in 6 mL of petroleum ether. The mixture was cleansed by a solid-phase extraction (SPE) cartridge silica, previously conditioned in petroleum ether; then the cartridge was rinsed with 12 mL of petroleum ether and underwent a flux of nitrogen for 10 min. Polyphenolic compounds were eluted with a mixture of methanol/distilled water (80/20; *v*/*v*) and subsequently with 8 mL of acetonitrile. After evaporation of the eluate at 50 °C using low pressure, the residue was dissolved with a mixture of methanol/distilled water (60/40; *v*/*v*) and filtered using a 0.45 pm nylon membrane. Polyphenol analysis was carried out at Lara-Spiral laboratory, using HPLC-mass spectrometry (HPLC-MS). 

#### 2.1.4. DPPH Free Radical Scavenging Activity

The 2, 2-diphenyl-1-picrylhydrazyl (C_18_H_12_N_5_O_6_; DPPH) assay was used to measure the free radical scavenging activity of seed oils. DPPH was solubilized in methanol and added to each oil sample. All samples were incubated in darkness for 30 min and the absorbance was read at 517 nm. The ascorbic acid (AA) and water were used as positive and negative controls, respectively. The DPPH free radical scavenging activity was calculated as follows:

DPPH free radical scavenging activity (%) = ((Absorbance negative control − Absorbance sample)/Absorbance negative control) × 100

#### 2.1.5. Ferric Reducing-Antioxidant Power (FRAP)

The FRAP test, which consists of reducing ferric iron (Fe^3+^) to ferrous iron (Fe^2+^), is used to determine the antioxidant potential of some compounds. The test was performed as described by Lim et al. [26]. Briefly, 0.4 mL of oils samples (prepared at different dilutions) were added to 1 mL of 0.2 M phosphate buffer (pH 6.6) and 1 mL potassium ferricyanide (1%) complex. The mixtures were incubated in darkness at 50 °C for 30 min. Then, 1 mL of trichloroacetic acid (TCA) solution (10%) was added to stop the reaction. After centrifugation at 3000g for 10 min, 1 mL of supernatant of each sample was mixed with 0.2 mL of 1% freshly prepared FeCl_3_ solution. The absorbance was measured at 700 nm. AA was used as a positive control. The FRAP of the oils was calculated as follows:

FRAP (%) = ((Absorbance sample/Absorbance blank) × 100)/Absorbance sample.

#### 2.1.6. Chelation Power on Ferrous (Fe^2+^) Ions

The chelating effect of the seed oils on ferrous ions was determined according to the method of Dinis et al. [27] with some modifications. Briefly, different concentrations of each oil were prepared at 1 mL and each sample was raised to 4.7 mL with distilled water. 100 μL of 2 mM FeCl_2_ solution was added and the samples were mixed. Then, 200 μL of 5 mM ferrozine was added to the mixture. After agitation, the samples were incubated at room temperature (RT) for 10 min and the absorbance was read at 562 nm. The iron-chelating power was determined as follows:

Iron-chelating power (%) = ((Absorbance negative control − Absorbance sample)/Absorbance negative control) × 100

### 2.2. In Vitro Study

#### 2.2.1. Cell Culture

Human neuroblastoma cells (SH-SY5Y) were cultured in Ham’s F12 GlutaMax medium (F12 medium; Gibco, Zaragoza, Spain) supplemented with 15% (*v*/*v*) heat-inactivated fetal bovine serum (FBS; Gibco, Zaragoza, Spain) and 1% antibiotics (penicillin, streptomycin) (Gibco, Zaragoza, Spain) in a humidified atmosphere containing 5% CO_2_. 

#### 2.2.2. Cell Treatments

7β-OHC was provided by Mohammad Samadi (University of Lorraine, Metz, France). A stock solution of 7β-OHC (800 μg/mL= 2 mM) was prepared as described by Ragot et al. [28]. Cells were seeded in 96, 24, or six-well plates. After 24 h, the growth medium was removed and cells were cultured in the absence or in the presence of 7β-OHC at various concentrations (10, 20, 30, 40, 50, and 60 µg/ mL) for 24 h. Hydrogen peroxide (H_2_O_2_, Sigma, Madrid, Spain) was used as a positive control of cytotoxicity. All experiments were performed using subtoxic conditions of 7β-OHC (able to induce mild oxidative stress and cell death).

Stock solutions of NSO and MTSO were prepared at 10% (*v*/*v*) in dimethyl sulfoxide (DMSO). After 24 h of culture, cells were incubated with 7β-OHC for 24 h with or without NSO or MTSO. Oils were added to the cells 2 h before the challenge with 7β-OHC. Trolox (synthetic polar analog of vitamin E, (Sigma, Madrid, Spain), was used as a positive control for cytoprotection. Treatment with 7β-OHC or H_2_O_2_ was maintained for 24 h except in the experiment of ROS measurement in which cells were treated for only 15 min to assess instantaneous ROS production.

#### 2.2.3. Cell Viability by MTT Assay

Methyl thiazolyl-diphenyl-Tetrazolium Bromide (MTT) assay evaluates cell survival due to its conversion into blue formazan by the succinate dehydrogenase [29]. SH-SY5Y cells were seeded at 25 × 10^3^ cells/well in a 96-well plate with 100 µL of culture medium and treated as described in the Section 2.2.2. 2 h before the end of the treatment, 10% (regarding cell medium volume) of MTT stock solution (5 mg/mL) was added to the cells. The medium was removed, and formazan was solubilized by adding 100 µL of DMSO (Sigma-Aldrich, Madrid, Spain), and the absorbance of each well was read at 540 nm and 650 nm (as reference) using an absorbance plate reader (BMG Labtech Fluostar Optima. Ortenberg, Germany). Data are expressed as the percentage of MTT reduction relative to the absorbance of untreated cells (controls).

#### 2.2.4. Cell Counting by Trypan Blue Exclusion Test

At the end of treatments, adherent and non-adherent cells were collected by trypsinization. Cells were counted with a hemocytometer under a light microscope in the presence of trypan blue (*v*/*v*) (Thermo Fisher Scientific, Barcelona, Spain), which stains non-viable cells in blue. Data are expressed as the total viable cells (× 10^3^)/well.

#### 2.2.5. Intracellular ROS Measurement by H_2_DCFDA Fluorescence Assay

The capacity of 7β-OHC to induce ROS production in SH-SY5Y cells was evaluated using 2′7′-dichlorofluorescein diacetate (H_2_DCFDA) fluorescence assay. This compound enters cells and is transformed into 2′7′-dichlorofluorescein (DCF) by the cell esterases and emit fluorescence when oxidized by ROS. SH-SY5Y cells were seeded at a density of 30 × 10^4^ cells/mL in a 24-well plate and then incubated for 24 h at 37 °C. Following incubation, the medium was removed; the cells were washed twice with Phosphate-Buffered Saline (PBS; pH 7.2) and incubated with a medium containing 10 µM of H_2_DCFDA for 30 min. After incubation, the culture supernatant was removed, and cells were incubated for 15 min with 7β-OHC (40 µg/mL) in PBS (pH 7.2). H_2_O_2_ was used as a positive control. Fluorescence was read at 485 ± 10 nm for excitation and 530 ± 12.5 nm for emission [30] using a fluorescence microplate reader (BioTek Synergy HTX, Winooski, VT, USA).

#### 2.2.6. Determination of the Enzymatic and Non-Enzymatic Antioxidants Levels

Total superoxide dismutase (SOD) activity was assessed in cells according to Beauchamp and Fridovich method [31]. The assay measures the ability of SOD to inhibit the nitroblue tetrazolium chloride (NBT) reduction. Briefly, cell lysate was added to the reaction mixture (50 mM in potassium phosphate buffer, pH 7.8), 0.1 mM EDTA, 13 mM L-methionine, 2 μM riboflavin, and 75 mM nitroblue tetrazolium (NBT). The developed blue color (formazan) in the reaction is proportional to SOD activity and was measured at 560 nm. Units of SOD activity are expressed as the amount of enzyme required to inhibit the reduction of NBT by 50%, and the activity is expressed as a percentage of controls.

Glutathione peroxidase (GPx) activity was measured as described by Flohe and Günzler [32]. The assay is based on glutathione (GSH) oxidation by GPx in the presence of 5-5′-Dithio-bis (2-nitrobenzoic acid) (DTNB). Cell lysates were incubated with 0.1 mM of GSH and PBS (50 mM, pH 7.8) for 5 min. The reaction was initiated by adding H_2_O_2_ (1.3 mM) and stopped by adding TCA (1%). After incubation at 4 °C for 30 min, the mixture was centrifuged at 1000× *g* for 10 min and the supernatant was transferred into new tube. DTNB was then added, and the absorbance was read at 412 nm. GPx activity is expressed as percentage of control cells.

Thiol (SH) groups play a potent role in the protection against free radicals. SH group levels were measured as described by Faure and Lafon [33]. Cell lysates were mixed with phosphate buffer (50 mM, PH = 8) and DTNB and incubated in the dark at RT for 15 min. The absorbance of the resulting product was read at 415 nm and data are expressed as µmol/mg of protein.

#### 2.2.7. Determination of Lipid Peroxidation Product Levels

Lipid peroxidation was assessed indirectly in SHSY5Y cells by measuring the production of malondialdehyde (MDA) and conjugated dienes (CD). The cell lysates were obtained by sonication followed by centrifugation at 20,000× *g* for 30 min. Protein content in cell lysate was determined by the Bradford method [34] using bovine serum albumin (BSA) as a standard.

The level of MDA was determined as described by Yoshioka et al. [35]. To precipitate the proteins, cell lysate was mixed with trichloroacetic acid (20%). Thiobarbituric acid (0.67%) was added later, and the mixture was incubated for 30 min at 95 °C. After cooling the mixture at RT, 4 mL of n-butanol was added, and the absorbance was measured at 530 nm using a spectrophotometer. MDA level was calculated using the molar extinction coefficient (1.56·10^−3^ mM^−1^·cm^−1^). Data are expressed in nmoles/mg of protein.

The CD level was determined as described by Esterbauer et al. [36]. The method is based on extracting lipids using a chloroform–methanol (2v:1v) mixture. The extracted lipids were redissolved in hexane for analysis, and CD concentration was determined using the molar extinction coefficient (2.52 × 104 M^−1^·cm^−1^). Data are expressed as µmoles of hydroperoxide/mg of protein.

#### 2.2.8. Determination of Protein Oxidation Products

Alongside lipids, proteins can also be damaged by ROS. The determination of carbonylated proteins (CP) level was determined spectrophotometrically as described previously [37]. Data are expressed as µmoles/mg of protein.

#### 2.2.9. Nitrotyrosination Study by Immunocytochemistry

SH-SY5Y cells were seeded on coverslips at 30 × 10^4^ cells/well in 24-wells plates. After treatment, cells were fixed with 4% paraformaldehyde (PFA) for 10 min. Cells were then permeabilized with 0.1% Triton X-100 (Sigma-Aldrich, Madrid, Spain) in 1X PBS for 10 min and blocked using a blocking buffer (2% BSA (Sigma-Aldrich, Madrid, Spain) in 1X PBS for 1 h at RT. Firstly, coverslips were incubated overnight at 4 °C with the primary antibody; anti-nitrotyrosine (1:200, Thermo Fisher Scientific, Barcelona, Spain) diluted in blocking buffer, then incubated in darkness with Alexa rabbit 488 conjugated secondary antibody (1:2000; Thermo Fisher Scientific, Barcelona, Spain), diluted in blocking buffer for 1h at RT. Cells were then stained with 4′,6-diamidino-2-phenylindole (DAPI) (1:1000; Thermo Fisher Scientific, Barcelona, Spain) for 15 min. Finally, coverslips were mounted on glass slides in 8 µl of Fluoromount (Southern Biotech, Birmingham, AL, USA).

#### 2.2.10. Caspase 3 Activation Study

SH-SY5Y cells were seeded on coverslips at 30 × 10^4^ cells/well in 24- well plates. After treatment, cells were fixed with PFA (4%) and permeabilized with 0.1% Triton X-100 in PBS at RT. Cells were blocked in 2% BSA at RT for 1 h then incubated overnight at 4 °C with 1:100 rabbit anti-cleaved Caspase-3 antibody (Cell Signaling). Cells were washed with PBS (3 × 5 min) and incubated with Alexa rabbit 488 conjugated secondary antibody (1:2000; Thermo Fisher Scientific, Barcelona, Spain) for 1 h at RT. Cells were rinsed (3 × 5 min) and stained with DAPI (1:1000; Thermo Fisher Scientific, Barcelona, Spain). Coverslips were mounted with Fluoromount (Southern Biotech, Birmingham, AL, USA). Digital images were taken with a Leica TCS SP confocal microscope with the Leica confocal software. Analysis and quantification were performed using Image J software.

### 2.3. Statistical Analysis

Statistical analysis was performed using SPSS version 22 (Statistical Package for Social Science, SPSS Inc., Chicago, IL, USA). Data were represented as mean ± standard deviation (SD). Analyses were carried out with T-test student and/or the Mann–Whitney U-test. Data were considered statistically different at a value of *p* ≤ 0.05.

## 3. Results

### 3.1. Biochemical Composition and Antioxidants Activities of NSO and MTSO

#### 3.1.1. NSO and MTSO Contents Analysis

The biochemical composition of NSO and MTSO (fatty acids, tocopherols, and polyphenols) is shown in Table 1.

The analysis of unsaturated fatty acids showed a relatively similar composition of NSO and MTSO except for α-linolenic acid (C18:3 n-3). In MTSO, both α-tocopherol and γ-tocopherol were detected (unlike NSO). α- tocopherol, a potent antioxidant, was the most abundant though (202 ± 17 mg/kg).

Polyphenols are known to have potent neuroprotective properties [15,16], thus polyphenols in NSO and MTSO were determined. Our results showed a high level of thymoquinone in NSO followed by vanillin, homovanillic acid, and quercetin. In MTSO, only quercetin and apigenin were detected (Table 1).

#### 3.1.2. Antioxidant Activities of NSO and MTSO

**Figure 1:**
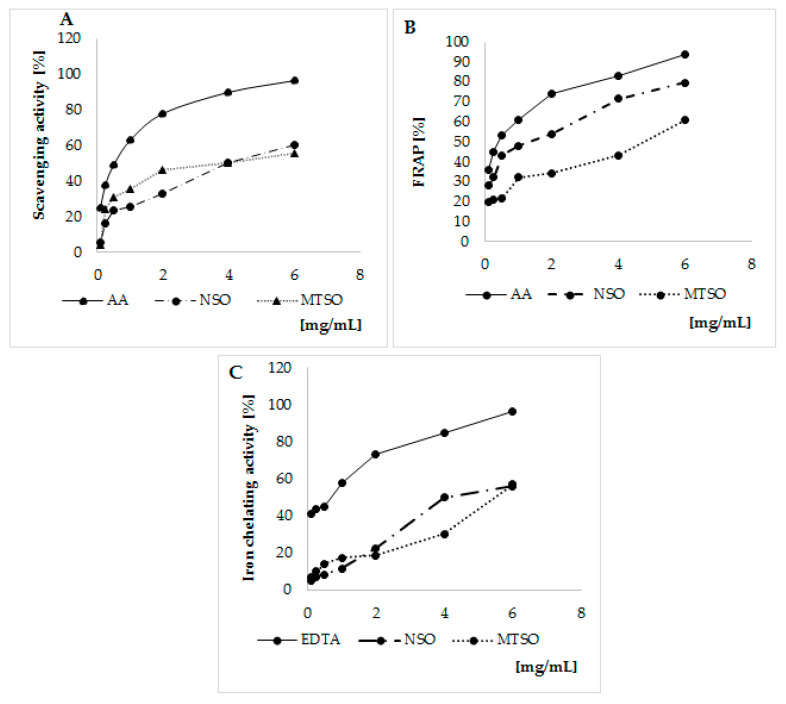
Antioxidant activities of NSO and MTSO. (**A**) DPPH assay. (**B)** FRAP assay. (**C**) Iron chelating Assay. NSO: Nigella seed oils; MTSO: Milk Thistle seed oils; EDTA: Ethylenediaminetetraacetic acid; AA: Ascorbic acid; FRAP: Ferric Reducing Antioxidant Power; DPPH 2,2-diphenyl-1-picrylhydrazyl.

The antioxidant activities of NSO and MTSO are represented in Figure 1.

Both NSO and MTSO exhibit free radicals scavenging activity as shown by DPPH test. Their activities were lesser than the AA (Figure 1A). To compare the scavenging activity of the oils, the half-maximal inhibitory concentration (IC50) (volume of seed oils required to lower the initial DPPH concentration by 50%) was determined from the dose–response curve (Table 2).

Our result indicates a free radical scavenging activity of NSO similar to MTSO. IC50 values were 4.11 ± 0.16 and 4.30 ± 0.33 (mg/mL), in NSO and MTSO, respectively.

The reducing power of each oil measured by FRAP was investigated along with AA used as standard reference (Figure 1B). NSO showed higher reducing power regarding MTSO. IC50 value were 1.85 ± 0.18 and 4.53 ± 0.23 (mg/mL) in NSO and MTSO, respectively (Table 2). In addition, a slightly higher iron chelating activity was observed in NSO treated cells compared to MTSO (Figure 1C and Table 2).

Although our results indicate potential antioxidant capacity of the two studied seed oils, NSO showed the highest antioxidant activities.

### 3.2. Evaluation of the Cytotoxicity and the Pro-Oxidative Activity of 7β-OHC

#### 3.2.1. The Effect of 7β-OHC on Cell Viability and Proliferation

**Figure 2:**
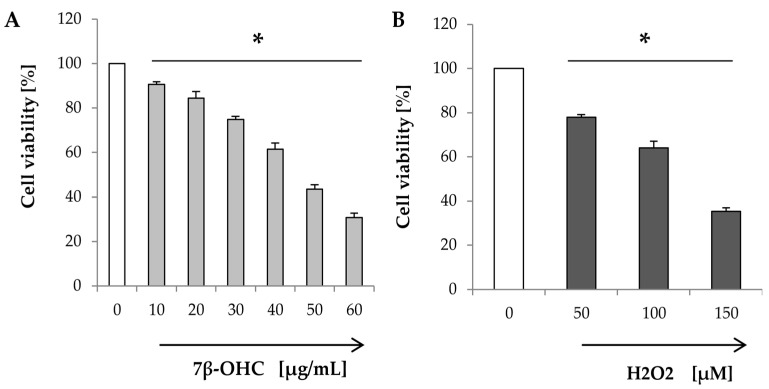
Effect of 7β-OHC on cell viability and proliferation. (**A**) MTT assay: Cells were incubated with different concentrations of 7β-OHC (10–60 µg/mL) for 24 h. (**B**) MTT assay: Cells were incubated with different concentrations of H_2_O_2_ (50–150 µM) for 24 h. Data are represented as mean ± SD. 7β-OHC: 7β-hydroxycholesterol; H_2_O_2_: Hydrogen peroxide. Statistical analyses were performed using the Mann–Whitney test or student t test. * Statistical differences were significant between the control and 7β-OHC or H_2_O_2_-treated cells (*p* < 0.05).

The MTT assay is widely used to study mitochondrial activity, cell viability, and /or cell proliferation. The effect of 7β-OHC (compared to H_2_O_2_) on cell viability is shown in Figure 2.

Our results showed that increasing concentrations of 7β-OHC were toxic in a dose-dependent manner (Figure 2A). Similar results were obtained with H_2_O_2_ (used as a positive control of toxicity) (Figure 2B).

In the presence of 7β-OHC (40 µg/mL), cell viability was significantly reduced to 61.53 ± 2.76 (%) compared to control. A comparable effect was obtained with H_2_O_2_ (100 µM).

Since 7β-OHC (40 µg/mL) induced a moderate toxicity (40%) in SH-SY5Y cells, this concentration was chosen in the rest of the study.

#### 3.2.2. The effect of 7β-OHC on Intracellular ROS Production

**Figure 3:**
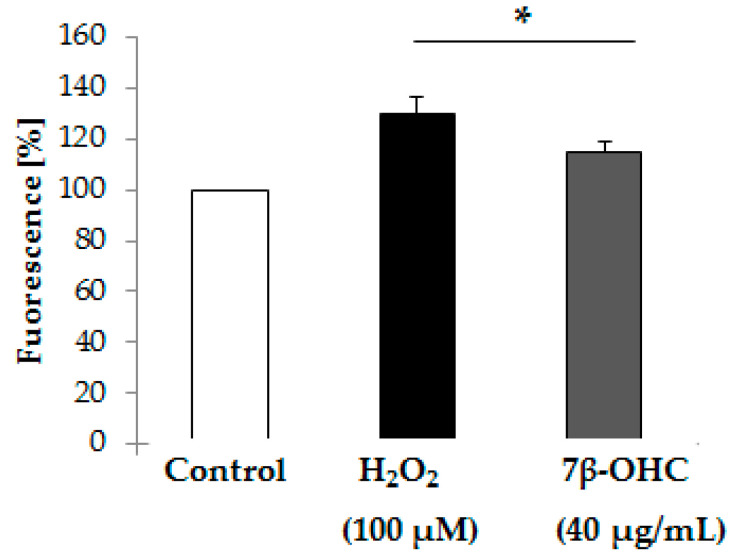
Effect of 7β-OHC on Intracellular ROS production by H_2_DCFDA fluorescence assay. Data are represented as mean ± SD. ROS: reactive oxygen species; H_2_DCFDA: 2′7′-dichlorofluorescein diacetate; DCF: 2′7′-dichlorofluorescein; 7β-OHC: 7β-hydroxycholesterol; H_2_O_2_: Hydrogen peroxide. Statistical analyses were performed using the Mann–Whitney test or student t test. * Statistical differences were significant between the control and 7β-OHC or H_2_O_2_-treated cells (*p* < 0.05).

Numerous studies reported pro-oxidative activities of oxysterols including 7β-OHC [37,38,39,40]. Thus, we intended to evaluate the ability of 7β-OHC (40 µg/mL) to induce intracellular ROS production in comparison with H_2_O_2_ (100 µM) using H_2_DCFDA fluorescence assay. Results are shown in Figure 3.

Our results indicate a significant increase in ROS production in 7β-OHC-treated cells, compared to control. The same result was obtained after exposing cells to H_2_O_2_ (100 µM). (Figure 3). These findings are in favor of the pro-oxidative activity of 7β-OHC.

#### 3.2.3. The Effect of 7β-OHC on Cellular Oxidative Stress

The effect of 7β-OHC on redox status was evaluated through the determination of the enzymatic and non-enzymatic antioxidants, lipid, and protein oxidation products. Results are represented in Tables 3–5.

Under the treatment with 7β-OHC (40 µg/mL), a significant decrease of GPx and SOD activities was observed (*p* < 0.05), compared to untreated and vehicle (EtOH)-treated cells. This may reflect either an alteration of the enzymatic antioxidant defense system in viable cells or the loss of the enzymatic activities due to cell death.

Thiol groups, known to play an important role in the protection against free radicals, were also found to be decreased in cells treated with 7β-OHC (Table 3). Our findings imply an insufficiency of the enzymatic and non-enzymatic antioxidant system defense regarding 7β-OHC.

Compared to untreated or vehicle-treated cells, lipid, and protein oxidation products (MDA, CD, and CP) were found at higher level in cells treated with 7β-OHC supporting thereby the pro-oxidative property of this oxysterol.

### 3.3. The Cytoprotective Study of NSO and MTSO

#### 3.3.1. The Effect of NSO and MTSO Associated with 7β-OHC on Cell Viability and Proliferation

**Figure 4:**
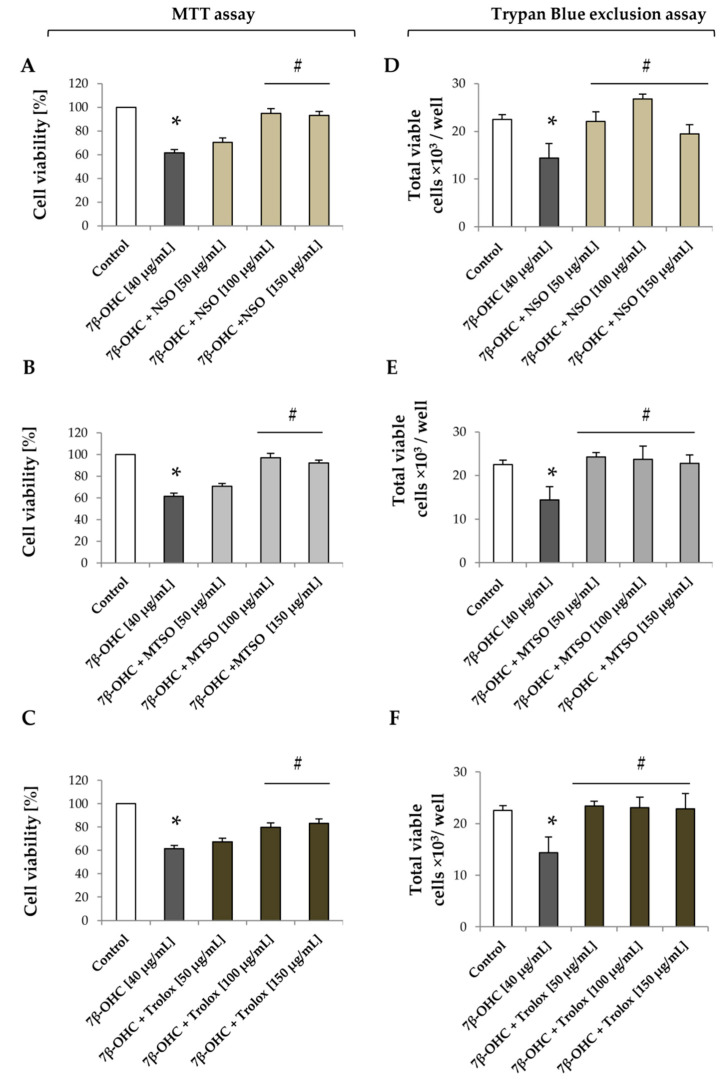
Effect of NSO and MTSO associated with 7β-OHC on cell viability and proliferation. (**A**) effect of NSO (50–150 µg/mL) associated with 7β-OHC (40 µg/mL) on cell viability and proliferation by the MTT assay. (**B**) effect of MTSO (50–150 µg/mL) associated with 7β-OHC (40 µg/mL) on cell viability and proliferation by the MTT assay. (**C**) effect of trolox (50–150 µg/mL) associated with 7β-OHC (40 µg/mL) on cell viability and proliferation by the MTT assay. (**D**) effect of NSO (50–150 µg/mL) associated with 7β-OHC (40 µg/mL) on cell viability and proliferation by the trypan blue exclusion assay. (**E**) effect of MTSO (50–150 µg/mL) associated with 7β-OHC (40 µg/mL) on cell viability and proliferation by the trypan blue exclusion assay. (**F**) effect of trolox (50–150 µg/mL) associated with 7β-OHC (40 µg/mL) on cell viability and proliferation by the trypan blue exclusion assay. Data are represented as mean ± SD. 7β-OHC: 7β-hydroxycholesterol; NSO: Nigella sativa seed oil; MTSO: Milk Thistle seed oil; MTT: Methyl thiazolyldiphenyl-Tetrazolium Bromide. Statistical analyses were performed using the Mann–Whitney test or student t test. * Statistical differences were significant between the control and or 7β-OHC-treated cells (*p* < 0.05). ^#^ Statistical differences were significant between 7β-OHC-treated cells and 7β-OHC + (NSO or MTSO) -treated cells (*p* < 0.05).

The effect of NSO and MTSO, associated with 7β-OHC, on cell viability and proliferation are shown in Figure 4.

Our results indicate a decrease in cells viability and proliferation (by MTT assay) under treatment with 7β-OHC (40 µg/mL) (Figure 4A). This observation was also confirmed by the trypan blue exclusion assay. Compared to untreated cells, the number of total viable cells was significantly reduced to 14 ± 3 × 103 cell /well in the presence of 7β-OHC (40 µg/mL) (Figure 4E). Interestingly, treatment with NSO, MTSO, or Trolox (used as a positive control) have restored cell viability and proliferation, especially at 100 µg/mL (Figure 4).

A similar protective effect was observed against H_2_O_2_-induced cytotoxicity (Figures in Supplementary Materials).

These data led us to assess further the mechanism by which both oils afford cytoprotection via the evaluation of oxidative stress biomarkers and the characterization of cell death.

#### 3.3.2. The Effect of NSO and MTSO Associated with 7β-OHC on the Enzymatic and Non-Enzymatic Cellular Antioxidants Defense System

The levels of enzymatic and non-enzymatic antioxidants in the presence of NSO and MTSO (associated or not with 7β-OHC (40 µg/mL)) are represented in Table 3.

Under the treatment with NSO and MTSO alone, GPx and SOD activities were relatively similar to those of untreated or vehicle (DMSO)-treated cells. In the presence of 7β-OHC, both GPx and SOD activities were decreased as described before. However, an increase in those activities was observed in 7β-OHC + NSO or MTSO -treated cells (Table 3). Our results showed also a higher enzymatic activity in 7β-OHC+MTSO (100 µg/mL)-treated cells regarding control. This could reflect an enhancement of cell proliferation. Although thiol groups were decreased in cells treated with 7β-OHC, neither NSO nor MTSO were able to modulate the production of these molecules.

#### 3.3.3. The Effect of NSO and MTSO Associated with 7β-OHC on the Lipid Peroxidation Levels

The effects of NSO and MTSO (associated or not with 7β-OHC) on the lipid peroxidation biomarkers levels are shown in Table 4.

No significant difference in MDA and CD levels was seen between control or vehicle (DMSO) and NSO or MTSO-treated cells. Although increased levels of those products were found in the presence of 7β-OHC (40 µg/mL), a marked reduction of MDA and CD concentration was observed in cells pre-treated with MTSO or NSO (100 μg/mL) (Table 4).

#### 3.3.4. The Effect of NSO and MTSO Associated with 7β-OHC on the Protein Oxidation and Nitrotyrosinantion

**Figure 5:**
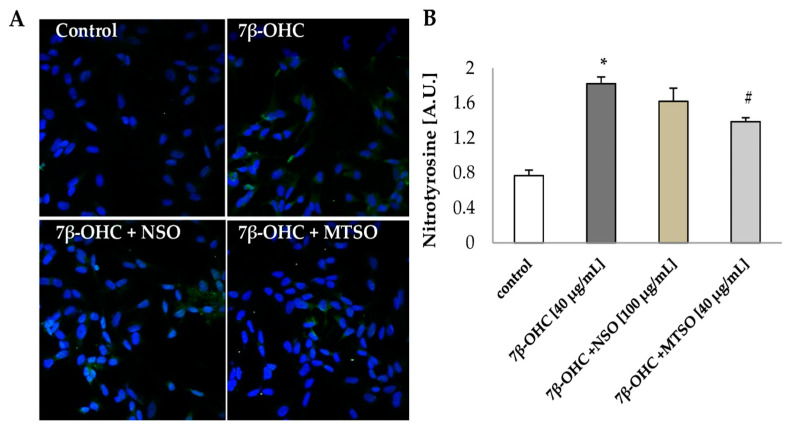
The cytoprotective effects of NSO and MTSO to proteins. (**A**) Confocal immunofluorescence microscopy observation of Nitrotyrosine (green fluorescence) with DAPI-stained nuclei (blue fluorescence) in SHSY5Y cells. (**B**) Nitrotyrosine quantification. Data are shown as mean ± SD. A.U: arbitrary units; 7β-OHC: 7β-hydroxycholesterol; NSO: Nigella sativa seed oil; MTSO: Milk Thistle seed oil. Statistical analyses were performed using the Mann–Whitney test or student t test. * Statistical differences were significant between the control and 7β-OHC-treated cells (*p* < 0.05). ^#^ Statistical differences were significant between the 7β-OHC-treated cells and 7β-OHC + (NSO or MTSO)-treated cells (*p* < 0.05).

To investigate the cytoprotective effects of NSO and MTSO on proteins; the levels of CP and nitrotyrosine were evaluated (Table 5 and Figure 5).

No significant difference in CP level was seen between control or vehicle (DMSO) and NSO or MTSO-treated cells. However, a significant increase in CP concentration was observed under treatment with 7β-OHC (40 µg/mL). The pre-treatment with seeds oils, especially with MTSO (100 μg/mL), showed a decrease of CP formation (Table 5), supporting further the cytoprotective activity of MTSO against oxidative damages.

The study of protein nitrotyrosination affords information on the production of reactive nitrogen species (RNS), especially peroxynitrite radical (ONOO−) [41]. In our study, 7β-OHC-treated cells showed a significant increase of nitrotyrosine level compared to control, indicating increased RNS production (1.82 ± 0.08 vs 0.77 ± 0.06 (A.U), respectively). Although no significant reduction of protein nitrotyrosination was seen under pre-treatment with NSO. Interestingly, a significant decrease of nitrotyrosine concentration was observed in cells pre-treated with MTSO compared to 7β-OHC-treated cells alone (*p* < 0.05) (Figure 5).

#### 3.3.5. NSO and MTSO Protects against Apoptosis Induced by 7β-OHC

**Figure 6:**
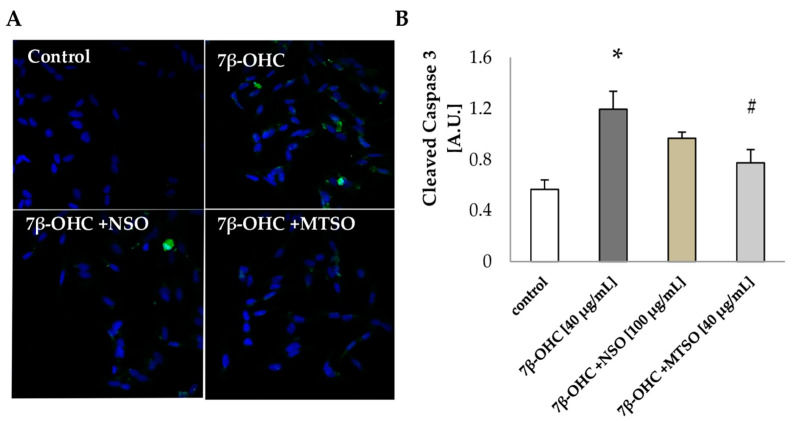
The cytoprotective effects of NSO and MTSO against cell death (**A**) Confocal immunofluorescence microscopy observation of cleaved caspase 3 (green fluorescence) with DAPI-stained nuclei (blue fluorescence) in SHSY5Y cells. (**B**) Cleaved caspase 3 quantification. Data are shown as mean ± SD. A.U: arbitrary units; 7β-OHC: 7β-hydroxycholesterol; NSO: Nigella sativa seed oil; MTSO: Milk Thistle seed oil. Statistical analyses were performed using the Mann–Whitney test or student t test. * Statistical differences were significant between the control and 7β-OHC-treated cells (*p* < 0.05). ^#^ Statistical differences were significant between the 7β-OHC-treated cells and 7β-OHC + (NSO or MTSO)-treated cells (*p* < 0.05).

Based on the MTT and trypan blue assays data, we investigated further the activation of the pro-apoptotic machinery by the detection of cleaved caspase 3 under the pre-treatment with NSO and MTSO. Results are shown in Figure 6.

Our findings indicate that 7β-OHC triggers apoptotic death in SHSY5Y cells via the activation of caspase 3 (*p* < 0.05). A marked reduction of cleaved-caspase 3 was obtained in cells pre-treated with MTSO (100 µg/mL) (*p* < 0.05). Although pre-treatment with NSO also showed a slight reduction of cleaved-caspase 3, the result was statistically non-significant.

The overall results suggest that NSO and, to a higher extent, MTSO can protect SH-SY5Y cells against 7β-OHC-induced oxidative damages and cell death.

## 4. Discussion

It is believed that oxysterols play a crucial role in the progression of chronic diseases including neurodegenerative diseases. These molecules can cross the blood–brain barrier and accumulate in the plasma membrane and inside the cell [42]. Some oxysterols, including 7β-OHC, exhibit pro-oxidative and pro-apoptotic properties [37,38,39,40].

In AD, PD, and HD conditions high levels of 7β-OHC were reported. Effective intervention strategies, including antioxidants therapies, could be a useful tool to oppose the pro-oxidative and pro-apoptotic activities of oxysterols.

In this line, our study was designed to investigate; (i) the ability of 7β-OHC to trigger oxidative stress and cell death in human neuroblastoma cells (SH-SY5Y) (ii) the capacity of Nigella sativa and Milk thistle seed oils (NSO and MTSO, respectively) to oppose 7β-OHC-induced side effects. Overall, our study establishes that 7β-OHC induces cell death and several oxidative damages on SH-SY5Y cells. Alongside, it probably suppresses cell proliferation, shown by the reduction of cell viability. NSO and, most importantly, MTSO were capable of attenuating 7β-OHC-induced toxicity. The cytoprotective effects of NSO and MTSO are likely related to ROS scavenging as well as the enhancement of cell proliferation.

The analysis of NSO and MTSO composition indicates that both oils represent good sources of essential fatty acids. In agreement with our results, studies reported that NSO and MTSO contain important amounts of linoleic, oleic, and α-linolenic acids [19,25]. In our study, a high concentration of thymoquinone, a potent antioxidant compound, was detected in NSO. This is in harmony with the previous studies [20,21,25]. Our results indicate the presence of both α- and γ- tocopherols in MTSO. Tocopherol profile is important to define the potential health benefits of vegetable oils and α- tocopherol is considered as the most biologically active isomer [43]. It is noteworthy that tocopherols and polyphenols can cross the blood–brain barrier. Moreover, these compounds showed potent neuroprotective properties [15,16], which is in favor of its consumption. In our study, the antioxidant activity of the oils, evaluated by DPPH, FRAP, and iron chelating assays, demonstrated an antioxidant potential of NSO and MTSO, which reinforced our interest to study their cytoprotective effects in vitro.

The cytotoxicity of 7β-OHC (40 µg/mL) on SH-SY5Y cells line was manifested by a reduction of cell viability and proliferation. Besides, numerous side effects were observed under treatment with this compound, consisting of apoptosis induction, ROS overproduction, impaired enzymatic and non-enzymatic antioxidants, and the increase of end products of lipid peroxidation, protein oxidation, and nitrotyrosination. Interestingly, the pre-treatment with NSO or MTSO (100 µg/mL) showed a marked attenuation of 7β-OHC-induced toxicity, implying a potential cytoprotective effect of NSO an MTSO.

In the literature, several studies reported the cytotoxicity of 7β-OHC in different cell lines including 158N and SK-N-BE [37,40,44,45]. Oxysterols are natural components of the human body. At low concentration, these molecules mediate many physiological functions within cells, such as enhancing the expression of inflammatory mediators (Interleukin 8) [46,47], modulating cholesterol homeostasis, and membrane permeability [48]. However, at higher concentration, some oxysterols can induce harmful effects. Indeed, anti-proliferative and pro-apoptotic activities of 7β-OHC were reported in different cell lines including murine oligodendrocytes (158N), monoblastic cell line (U937), and human liver cancer cell line (HepG2) [40,47,49,50,51]. In our study, an induction of apoptotic cell death by 7β-OHC was also observed. Besides, a reduction of cell viability and proliferation was seen under treatment with this compound, which is in harmony with the previous studies.

It is noteworthy that some oxysterols exhibit pro-oxidant property. Under treatment with 7β-OHC, high level of lipid and protein oxidation products (MDA, CD, and CP) as well as an increase of antioxidant enzymatic activities was reported [37,45]. These data are in partial agreement with our results. In the literature, few papers reported the effect of oxysterols, especially 7β-OHC, on the antioxidant enzymes activities. In addition, results are disparate. While in vitro studies suggested an increase of the antioxidant enzymes activities, under treatment with oxysterols, [37,45], a recent in vivo study reported a decrease of these activities [52]. In rat red blood cells exposed to different oxysterols (5α,6α-Epoxyphytosterols and 5α,6α-Epoxycholesterol), a significant decrease of the antioxidant enzymes activities (GPx and SOD but not catalase) was observed [52]. Besides, it has been reported that increased levels of oxysterols (25-hydroxy-, 7α-hydroxy-, and 7-ketocholesterol) and MDA were associated with the decrease of catalase and SOD activities in rats exposed to H_2_O_2_ [53]. However, it is difficult to conclude whether the deficiency in enzymatic activity was directly triggered by oxysterols or H_2_O_2_. In the present study, the decrease of antioxidant enzyme activities could be a cause, but also a consequence, of the accumulated ROS and RNS. In fact, nitrotyrosine detection is believed to be a biomarker of RNS generation and protein damages [54,55,56]. The protein–tyrosine–nitration can affect protein structure and function leading to macromolecules damages such as inactivation of certain enzymes including catalase and MnSOD [57,58]. Therefore, we can suggest that the loss of SOD and GPx activities in our study is caused by the increase of protein nitrotyrosination observed in 7β-OHC-treated cells. On the other hand, under physiological conditions, ROS are removed by the antioxidant system, but probably during mild and prolonged oxidative stress, the ROS concentration in cells reaches a threshold that can trigger downregulation of the antioxidant enzymes.

As reported belong, NSO and MTSO showed cytoprotective effects against the cytotoxicity mediated by 7β-OHC. Both oils, especially MTSO, were able to reduce lipid and protein oxidation, inhibit caspase 3 activation, and improve the antioxidant enzyme activities. In addition, these oils enhanced SH-SY5Y cells proliferation. In the literature, NSO and MTSO are known to have antioxidant activities, since these oils contain several bioactive components including polyphenols and tocopherols [20,21,22]. As such, milk thistle extract was reported to promote differentiation and neuronal survival as well as protects rat hippocampus neurons against cell death [23], which is in harmony with our results suggesting cytoprotective effects of NSO and especially MTSO. In fact, numerous studies indicate that molecules such as α-tocopherol [40], polyphenols including quercetin [59,60], dimethyl fumarate [61], docosahexaenoic acid [40,44,62], and oleic acid [62] can prevent the toxicity of 7KC induced in oligodendrocytes, microglial, and neuronal cells. Similarly, some studies reported an attenuation of the cytotoxicity of 7β-OHC by docosahexaenoic acid, α-tocopherol, and biotin [40,45]. We suggest that the bioactive molecules present in MTSO and NSO, such as thymoquinone, α-tocopherol, quercetin, and/or oleic acid are responsible, at least in part, for the cytoprotection against 7β-OHC-induced toxicity on SH-SY5Y cells.

## 5. Conclusions

Summarizing, 7β-OHC showed a complex of damages on SH-SY5Y cells, triggered by its anti-proliferative, pro-oxidative, and pro-apoptotic activities. Our study supports the contribution of oxysterols and especially, 7β-OHC, in the development cellular dysfunctions associated with chronic diseases including neurodegenerative disorders. NSO and especially MTSO were able to protect cells against 7β-OHC-induced toxicity. This study exhibits some limitations and opens several perspectives. In fact, the use of differentiated cells or primary culture would be considered a better model for neuronal cells, that mimic better physiological conditions. In addition, the use of purified bioactive molecules or mixtures of molecules to protect cells against oxysterols side effects would give them an advantage in considering therapeutic applications.

## Figures and Tables

**Table 1:** Fatty acids, tocopherols, and polyphenols contents in NSO and MTSO.

	NSO	MTSO
**Fatty acid content (% of total Fatty acids)**		
Ʃ unsaturated fatty acids	82.85 ± 0.14	83.83 ± 4.26
Oleic acid C18:1n-9	22.76 ± 0.02	21.39 ± 0.02
Linoleic acid C18:2n-6	54.56 ± 0.14	56.77 ± 0.57
α-Linolenic acid C18:3n-3	1.54 ± 0.02	0.49 ± 0.01
**Tocopherol content (mg/kg)**		
γ- Tocopherol	undetectable	92.00 ± 7.00 (220.88 ± 16.78 μmol/L)
α-Tocopherol	undetectable	202.00 ± 17.00 (468.67 ± 39.44 μmol/L)
Total Tocopherols Content	undetectable	274.00 ± 24.00
**Polyphenol content (mg equivalent quercetin/kg of oil)**		
Thymoquinone	7.00 ± 0.58	Undetectable
Vanillin	2.30 ± 0.06	Undetectable
Homovanillic Acid	1.90 ± 0.00	Undetectable
Quercetin	1.30 ± 0.02	1.20 ± 0.04
Apigenin	undetectable	0.90 ± 0.10

Data are represented as mean ± SD. NSO: Nigella seed oils; MTSO: Milk Thistle seed oils.

**Table 2:** IC50 values of DPPH, FRAP, and iron chelating activity of NSO and MTSO.

		IC50 Values (mg/mL)	
Samples	DPPH	FRAP	Iron Chelating
NSO	4.11 ± 0.16	1.85 ± 0.18	4.86 ± 0.11
MTSO	4.30 ± 0.33	4.53 ± 0.23	5.75 ± 0.24
EDTA (standard)	−	−	0.60 ± 0.09
AA (standard)	0.81 ± 0.27	0.41 ± 0.16	−

Data are represented as mean ± SD. IC50: Half-maximal inhibitory concentration; NSO: Nigella seed oils; MTSO: Milk Thistle seed oils; EDTA: Ethylenediaminetetraacetic acid; AA: Ascorbic acid; FRAP: Ferric Reducing Antioxidant Power; DPPH 2,2-diphenyl-1-picrylhydrazyl.

**Table 3:** Levels of enzymatic and non-enzymatic antioxidants under pre-treatment with NSO or MTSO.

	SOD (% Control)	GPx (% Control)	Thiols (µmol/mg of Protein)
Control	100 ± 0.00	100 ± 0.00	0.95 ± 0.01
(EtOH 0.2%)	97.85 ± 0.76	91.18 ± 9.36	0.92 ± 0.14
(DMSO 0.4%)	98.17 ± 2.34	98.25 ± 4.18	0.97 ± 0.09
(EtOH + DMSO)	97.88 ± 1.50	99.18 ± 9.28	0.95 ± 0.01
NSO (100 µg/mL)	99.30 ± 4.18	97.41 ± 5.40	0.94 ± 0.02
MTSO (100 µg/mL)	101.47 ± 2.78	96.81 ± 1.14	0.98 ± 0.07
7β-OHC (40 µg/mL)	34.73 ± 1.43 *	56.42 ± 5.25 *	0.48 ± 0.08 *
7β-OHC + NSO (100 µg/mL)	47.77 ± 0.89 #	75.65 ± 5.10	0.45 ± 0.08
7β-OHC + MTSO (100 µg/mL)	109.63 ± 7.73 #	106.26 ± 10.30 #	0.59 ± 0.04

Data are represented as mean ± SD. 7β-OHC: 7β-hydroxycholesterol; SOD: superoxide dismutase; GPx: glutathione peroxidase; NSO: Nigella sativa seed oil; MTSO: Milk Thistle seed oil; DMSO: dimethyl sulfoxide; EtOH: Ethanol. Statistical analyses were performed using the Mann–Whitney test or student t test. * Statistical differences were significant between the control and vehicle (EtOH) or 7β-OHC-treated cells (*p* < 0.05). ^#^ Statistical differences were significant between 7β-OHC-treated cells and 7β-OHC + (NSO or MTSO) -treated cells (*p* < 0.05).

**Table 4:** Levels of lipid peroxidation biomarkers under pre-treatment with NSO or MTSO.

	MDA (µmol/mg of Protein)	CD (µmol/mg of Protein)
Control	68.09 ± 3.43	0.55 ± 0.83
(EtOH 0.2%)	70.05 ± 5.70	0.40 ± 0.10
(DMSO 0.4%)	73.43 ± 1.11	0.54 ± 0.04
(EtOH + DMSO)	71.32 ± 0.43	0.56 ± 0.04
NSO (100 µg/mL)	69.96 ± 2.60	0.59 ± 0.03
MTSO (100 µg/mL)	70.93 ± 2.38	0.44 ± 0.01
7β-OHC (40 µg/mL)	99.39 ± 2.50 *	0.89 ± 0.07 *
7β-OHC + NSO (100 µg/mL)	79.64 ± 1.60 ^#^	0.52 ± 0.12 ^#^
7β-OHC + MTSO (100 µg/mL)	75.02 ± 0.90 ^#^	0.68 ± 0.19

Data are the mean ± SD. 7β-OHC: 7β-hydroxycholesterol; MDA: malondialdehyde; CDs: conjugated dienes; NSO: Nigella sativa seed oil; MTSO: Milk Thistle seed oil; DMSO: dimethyl sulfoxide; EtOH: Ethanol. Statistical analyses were performed using the Mann–Whitney test or student t test. * Statistical differences were significant between the control and vehicle (EtOH) or 7β-OHC-treated cells (*p* < 0.05). ^#^ Statistical differences were significant between 7β-OHC-treated cells and 7β-OHC + (NSO or MTSO) -treated cells (*p* < 0.05).

**Table 5:** Levels of protein oxidation biomarker under pre-treatment with NSO or MTSO.

	CP (µmol/mg of Protein)
Control	0.13 ± 0.01
(EtOH 0.2%)	0.12 ± 0.02
(DMSO 0.4%)	0.15 ± 0.01
(EtOH + DMSO)	0.12 ± 0.02
NSO (100 µg/mL)	0.13 ± 0.01
MTSO (100 µg/mL)	0.12 ± 0.02
7β-OHC (40 µg/mL)	0.39 ± 0.04 *
7β-OHC + NSO (100 µg/mL)	0.28 ± 0.03
7β-OHC + MTSO (100 µg/mL)	0.17 ± 0.02 ^#^

Data are represented as mean ± SD. 7β-OHC: 7β-hydroxycholesterol; CP: carbonylated proteins; NSO: Nigella sativa seed oil; MTSO: Milk Thistle seed oil; DMSO: dimethyl sulfoxide; EtOH: Ethanol. Statistical analyses were performed using the Mann–Whitney test or student t test. * Statistical differences were significant between the control and vehicle (EtOH) or 7β-OHC-treated cells (*p* < 0.05). ^#^ Statistical differences were significant between 7β-OHC-treated cells and 7β-OHC + (NSO or MTSO) -treated cells (*p* < 0.05).

## Data Availability

Data supporting the findings of this study are available from the first author (Souha Hammouda) on request.

## Supplementary Materials

The following are available online at https://www.mdpi.com/article/10.3390/biom11060797/s1, Figure S1: Vehicle controls for cell viability by MTT assays; Figure S2: NSO and MTSO concentration curves by MTT assays; Figure S3: Vehicle controls for cell viability by trypan blue test; Figure S4: Effect of NSO and MTSO associated with H_2_O_2_ on cell viability and proliferation by MTT assay (A) Effect of NSO (50–150 µg/mL) associated with H_2_O_2_ (100 µM) on cell viability and proliferation by MTT assay. (B) Effect of MTSO (50–150 µg/mL) associated with H_2_O_2_ (100 µM) on cell viability and proliferation by MTT assay. (C) Effect of trolox (50–150 µg/mL) associated with H_2_O_2_ (100 µM) on cell viability and proliferation by MTT assay.
